# Allelic Variations in Vernalization (*Vrn*) Genes in *Triticum* spp.

**DOI:** 10.3390/genes15020251

**Published:** 2024-02-17

**Authors:** Sanaz Afshari-Behbahanizadeh, Damiano Puglisi, Salvatore Esposito, Pasquale De Vita

**Affiliations:** 1 Research Centre for Cereal and Industrial Crops (CREA-CI), CREA—Council for Agricultural Research and Economics, SS 673 Meters 25 200, 71122 Foggia, Italy; sanaz.afshari@unifg.it (S.A.-B.); damiano.puglisi@crea.gov.it (D.P.); 2 Department of Agriculture, Food, Natural Science, Engineering, University of Foggia, Via Napoli 25, 71122 Foggia, Italy; 3 National Research Council of Italy, Institute of Biosciences and BioResources, Research Division Portici (CNR-IBBR), 80055 Portici, Italy

**Keywords:** ploidy, wheat, durum wheat, allelic variations, copy number variations

## Abstract

Rapid climate changes, with higher warming rates during winter and spring seasons, dramatically affect the vernalization requirements, one of the most critical processes for the induction of wheat reproductive growth, with severe consequences on flowering time, grain filling, and grain yield. Specifically, the *Vrn* genes play a major role in the transition from vegetative to reproductive growth in wheat. Recent advances in wheat genomics have significantly improved the understanding of the molecular mechanisms of *Vrn* genes (*Vrn-1*, *Vrn-2*, *Vrn-3*, and *Vrn-4*), unveiling a diverse array of natural allelic variations. In this review, we have examined the current knowledge of *Vrn* genes from a functional and structural point of view, considering the studies conducted on *Vrn* alleles at different ploidy levels (diploid, tetraploid, and hexaploid). The molecular characterization of *Vrn-1* alleles has been a focal point, revealing a diverse array of allelic forms with implications for flowering time. We have highlighted the structural complexity of the different allelic forms and the problems linked to the different nomenclature of some *Vrn* alleles. Addressing these issues will be crucial for harmonizing research efforts and enhancing our understanding of *Vrn* gene function and evolution. The increasing availability of genome and transcriptome sequences, along with the improvements in bioinformatics and computational biology, offers a versatile range of possibilities for enriching genomic regions surrounding the target sites of *Vrn* genes, paving the way for innovative approaches to manipulate flowering time and improve wheat productivity.

## 1. Introduction

Wheat species (*Triticum* spp.) are classified based on ploidy level into diploids (2n = 2x = 14), tetraploids (2n = 4x = 28), and hexaploids (2n = 6x = 42), including wild and domesticated species as well as landraces, and old and modern elite cultivars [1,2].

The species of the *Triticum* genus derive from spontaneous hybridization and domestication events with closely related goat-grass species (*Aegilops* spp.). In an allopolyploid wheat, the genomes of the diploid progenitors become homoeologous subgenomes, because multiple paralogues (copies of a gene) are found at each locus. Many genes in the subgenomes are expected to be similar in sequence and regulation, but others might be divergent. Polyploidy appears to have occurred spontaneously in the *Triticeae* tribe in different periods of history, making the *Triticum* genus an exceptional lineage for investigating plant allopolyploidy, evolutionary dynamics, agricultural domestication processes, genetic diversity, and the mechanisms governing adaptation to diverse local environments or stress conditions [1]. Several studies demonstrated the important role played by polyploidy in the diversification of plant species, gene evolution, and the domestication processes in crops [3,4,5,6]. In wheat, domestication led to the appearance of soft glumes, nonfragile rachis, and free-wheat characteristics, increasing the diversity of wheat grains to end uses, and improving grain yield and adaptability to diverse environmental conditions [7,8]. Additional traits such as grain yield, shape and size of the seed, plant height, and flowering time were modified during domestication and by the subsequent breeding process [9]. Specifically, the spread of domesticated wheat originating from the Fertile Crescent has necessitated adaptation to new environments, facilitated by favorable alleles at critical genetic loci [10]. These traits provided allohexaploid bread wheat a competitive advantage in different environmental contexts. Bread wheat (*T. aestivum*) is generally more salt-tolerant than its tetraploid progenitor emmer (*T. Turgidum* subsp.) [11] and it has been associated with adaptation to cold stress [12,13]. However, among all these traits, the change in heading date and/or flowering time was one of the key traits allowing wheat to spread to new regions. The control of flowering time is crucial for the adaptation of the crop to different climatic conditions and has a significant impact on grain yield in *Triticeae* [14,15]. The regulation of flowering time is primarily determined by three groups of loci, of which two interact with environmental conditions [16]. Vernalization genes (*Vrn*) control the requirement for a cold period to transition from the vegetative to the reproductive phase, whereas photoperiod genes (*Ppd*) determine the response to day length (Distelfeld et al. [16]). The third group of loci is known as ‘narrow-sense earliness’ or ‘earliness per se’ (*Eps*), reflecting its independent role from vernalization and photoperiod [17]. *Vrn* genes play crucial roles in controlling growth habits and they represent the most important adaptative strategy to postpone heading after the winter stage, preventing frost damage [18,19,20]. The identification of *Vrn* genes has contributed to the classification of wheat cultivars into winter growth habit, in which exposure to cold temperatures is required to induce flowering, spring growth habit, which does not require exposure to cold temperatures to induce flowering, and intermediate growth habit [16,19,21,22,23]. Recently, a dozen functional and non-functional *Vrn* alleles have been identified in different wheat species, revealing their different contribution to wheat phenology. Identifying variation in these genes is a key point in controlling wheat phenology and satisfying the growing conditions prevalent in each agro-environment.

However, to better exploit the new information and define new phenological models of wheat, it is necessary to share with the scientific community the classification of the known allelic variants of the *Vrn* genes, highlighting ambiguous cases of nomenclature, which should be resolved.

Considering the above reasons, in this review, we first describe the main features and functions of *Vrn* genes and alleles, named according to Boden et al. [24], and then we summarize recent comprehensive studies of *Vrn* alleles at different levels of wheat ploidy that could be of great support in developing resilient wheat cultivars better adapted to higher or lower latitudes and different climatic regions. Furthermore, the review emphasizes the need for innovative genomic tools to effectively discriminate allelic variants, a critical obstacle in advancing the phenotypic characterization of individual alleles.

## 2. Characteristics and Functions of *Vrn* Genes

To date, four main *Vrn* genes have been described in wheat (*Vrn-1*, *Vrn-2*, *Vrn-3*, and *Vrn-4*) [19,25,26,27].

The *Vrn-1* gene encodes a *MADS-box* transcription factor (TF) that is homologous to *APETALA1* (*AP1*) in *Arabidopsis* and plays a crucial role in the vernalization response by regulating the transition of the vegetative shoot apical meristem to the reproductive phase [19,28,29,30,31]. The *Vrn-1* gene was also named *WAP1* by Trevaskis et al. [31], who reported that *Vrn-1* was strongly expressed in spring wheat, moderately expressed in semi-spring wheat, and not expressed in winter wheat. Bread wheat harbors three copies of *Vrn-1* (*Vrn-A1*, *Vrn-B1*, and *Vrn-D1*) located in the middle of the long arms of homoeologous chromosome 5 [19,32,33,34,35], in the same regions where the two copies of durum wheat (*Vrn-A1* and *Vrn-B1*) have been mapped [36,37].

The *Vrn-2* gene plays a crucial role as a flowering repressor, accelerating wheat flowering [19,25]. The *Vrn-A2* locus includes two tandemly duplicated CCT domain genes (*CONSTANS*, *CO-like*, and *TOC1*), namely *ZCCT1* and *ZCCT2*. Both are repressed during vernalization under long days [25], enhancing the activation of *Vrn-1* and promoting flowering [38]. In diploid and tetraploid wheat, the *Vrn-2* gene consists of two homoeologous copies, namely *Vrn-A2* and *Vrn-B2* [25], mapping on chromosomes 5AL and 4BL, respectively. *Vrn-A2* probably translocated from 4AL to 5AL in hexaploid wheat [39]. Indeed, *Vrn*-*B2* was mapped on 4BL but not 5BL, although highly similar sequences were also reported on chromosomes 2BS, 4BS, and 5DL, suggesting that duplication, deletion, and translocation events have impacted the *Vrn-B2* locus across different hexaploid wheat varieties [39]. *Vrn*-*D2* was detected in a genome contig sequence from 4DL but not from 5DL, suggesting that no translocations occurred between 4DL and 5DL in hexaploid wheat [39]. One or more homoeologous copies might also exist for *Vrn-D2* on chromosome 4D, although further analysis is needed to clarify its genomic position in bread wheat [16,40].

*Vrn-3* produces a mobile protein that functions as a flowering activator [26] and, as homologous to *FLOWERING LOCUS T* (*FT*) of *Arabidopsis* [26,41], is induced by long days, further accelerating reproductive apex development [26,38,42]. In hexaploid wheat, the three copies of *Vrn-3* (*Vrn-A3*, *Vrn-B3*, and *Vrn-D3*) were mapped on chromosomes 7A, 7B, and 7D, respectively [26,43,44,45,46]. Yan et al. [26] first suggested that *Vrn-3* was linked to a gene similar to *Arabidopsis FT*; the latter was then proposed by Tamaki et al. [47] as the candidate for *Florigen*, a locus encoding the mobile signal that induces floral initiation at the shoot apex [47]. *FT* encodes a poly ethanol amine binding protein (PEBP) [48,49], a class of proteins implicated in cellular signaling [50,51]. In *Arabidopsis*, *FT* expression increases in leaves when plants are exposed to long days [48,49], allowing its protein to be transported to the shoot apex to promote flowering [52]. Also, in wheat and barley, the expression of *FT* is induced by long days and promotes flowering [53].

*Vrn-4* encodes a *MADS-box* TF very similar to *Vrn-1* but, unlike *Vrn-1*, *Vrn-2*, and *Vrn-3*, its function has not yet been well clarified [54,55]. *Vrn-D4* probably operates upstream of the positive regulatory feedback loop connecting *Vrn-1*, *Vrn-2*, and *Vrn-3* [54,56]. It is located on the centromeric region of chromosome 5D in hexaploidy wheat [44] and it originated by the insertion of a segment on chromosome5AL, carrying the *Vrn-1* gene (~290-kb), into the proximal region of chromosome 5D [27]. The insertion of the 5AL region includes a copy of *Vrn-A1* with distinctive mutations in its coding and regulatory regions [27]. This insertion is almost fixed in the ancient wheat from South Asia [27], especially in India and nearby regions [57,58]. The gene was found for the first time in a wheat variety of Australian origin (i.e., Gabo) [59], which probably inherited it from an Indian variety (i.e., Muzaffarnagar) [60]. *Vrn-4* was mainly found at a higher frequency among wheat accessions of the ancient subspecies *T. aestivum* subsp. *sphaerococcum* from South Asia [58], a subspecies with an increased differentiation in the centromeric region of chromosome 5D, indicating that *Vrn-4* likely played a role in local adaptation through positive selection [27]. The centromeric region of homoeologous group 5 includes the *TaVIL1* gene [55], which encodes a homolog of the *Arabidopsis VIL1* (*Vernalization Insensitive 3 VIN3*-*like 1*). This gene, together with *VIN3*, is crucial for the epigenetic memory of vernalization and plays a role in the photoperiodic regulation of flowering time in *Arabidopsis* [61]. Taking into account its similarity with the *Arabidopsis VIL1* gene, its centromeric location, and its increased expression during vernalization, the wheat *TaVIL*-*D1* gene was previously considered as a potential candidate gene for *Vrn*-*D4* [55].

## 3. Allelic Variations within *Vrn* Genes at Different Ploidy Levels

**Figure 1:**
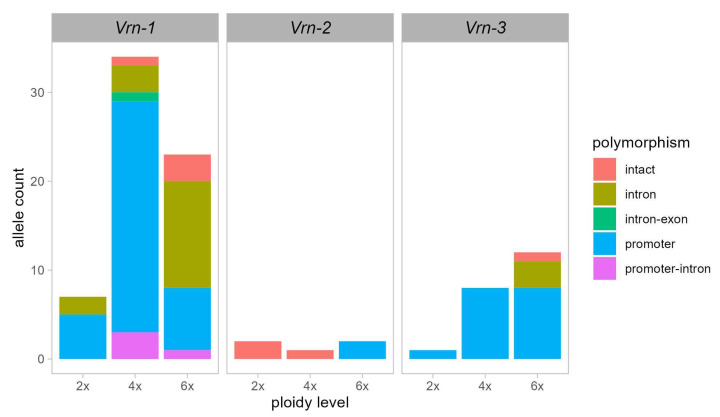
Survey of natural variations in *Vrn-1*, *Vrn-2*, and *Vrn-3* genes identified in the literature for each ploidy level: diploid (2x), tetraploid (4x), hexaploid (6x). Different colors represent the number of intact and natural allele variations in the promoter, intron, and both promoter and intron/exon. The *Vrn-4* gene was excluded due to a lack of polymorphisms (alleles). This graph was created using Tables S1–S3 (Supplementary Materials).

Figure 1 shows the number of alleles investigated in this review, with about 100 alleles examined, including 69 from *Vrn-1*, 6 from *Vrn-2* and 23 from *Vrn-3*. The highest number of alleles was identified in the promoter region (about 60), followed by the intron region (about 20) (Figure 1; Tables S1–S3).

Natural variation in vernalization requirements in temperate cereals is strongly associated with mutations at *Vrn-1*, *Vrn-2*, *Vrn-3*, and *Vrn-4* genes [16,19,25,26,27]. Spring and facultative growth habits harbour one or more dominant alleles at the *Vrn-1*, *Vrn-3*, or *Vrn-4* loci, while winter wheat harbour dominant allele(s) at the *Vrn-2* locus but recessive alleles at the other three loci [54]. Molecular analysis of *Vrn* loci allowed the scientific community to establish that multiple dominant alleles of these loci emerged through mutations occurring in two key regulatory regions: the promoter and the first intron. Specifically, *Vrn-A1* predominately carries mutations in the promoter region, whereas *Vrn-B1* and *Vrn-D1* predominately harbor deletions within the first intron [56,62,63,64]. In addition, copy number variations (CNV) at one of the *Vrn-1* loci were also reported in bread wheat by Diaz et al. [65] and Strejčková et al. [63].

### 3.1. Allelic Variation of Vrn-1 at the Promoter Level

**Figure 2:**
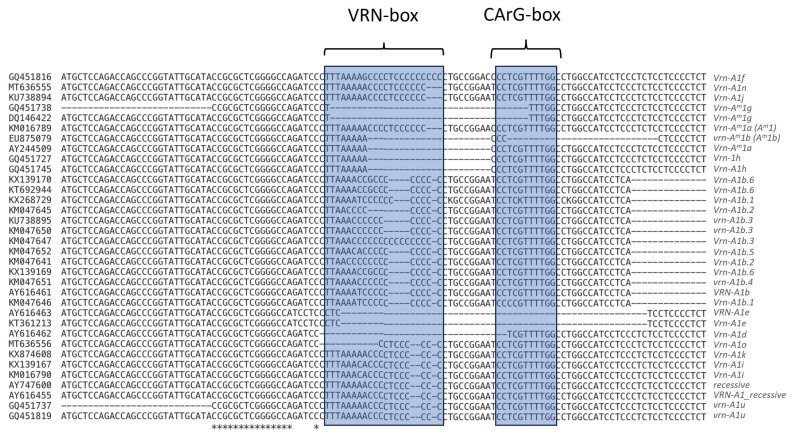
Multiple alignments of *Vrn-A1* alleles carrying mutations in the promoter region. For each nucleotide sequence, the deposited GenBank code and the name of the allele are specified. The VRN-box and CArG-box are highlighted in blue.

**Figure 3:**
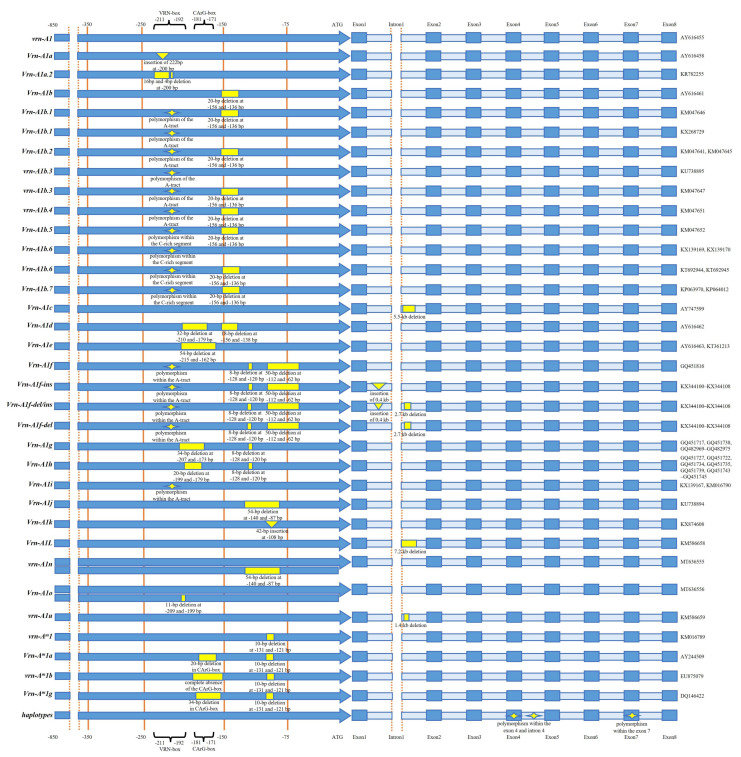
Schematic representation of natural variations identified within *Vrn-A1*. The figure depicts different polymorphism types identified in the literature not based on ploidy level. Different alleles are represented by different shapes: the star denotes polymorphism, rectangle deletion, and triangle insertions at the promoter, intron and/or exon regions. For each allele, the deposited GenBank code are specified on the right, while the corresponding references are available in Table S1. Haplotypes indicate the presence of different polymorphisms at exon-4 (*Ex4C*/*Ex4T*), intron-4 (*Ex4C.s*, *Ex4C.m*, *Ex4C.f*, and *Ex4C.sph*), and exon-7 (*Ex7C*/*Ex7T*) [74].

Genetic variations at the promoter level may significantly impact *Vrn-A1* expression and regulation [66] (Figure 1, Figure 2 and Figure 3; Table S1). Thus, understanding their diversity in genotypes with different ploidy levels can provide a valuable resource to further investigate the genetic basis of FT regulation in wheat. The promoter of *Vrn-1* is considered a repertoire of regulatory elements, of which CArG-box, VRN-box, and ACGT-motif are the most studied [66]. VRN-box is characterized by a 16 bp region (“TTAAAAACCCCTCCCC”) and is considered the most influential on the “winter-spring” growth habit [56], whereas CArG-box (a common binding site for MADS-box) is not critical, since genotypes with a fully deleted CArG-box region show a preserved vernalization machinery [62,67]. Distinct novel genetic variations have been revealed to be situated within the regulatory region of *Vrn-A1* (Figure 2).

*Vrn-A1a* stands out as one of the most significant and potent spring alleles [36]. It has a duplicated promoter region carrying characteristic foldback elements. The two fragments differed from the recessive *vrn-A1* allele by the insertion of a 222 bp foldback element in the larger fragment and a 131 bp foldback element in the smaller one [36]; it was reported that *Vrn-A1a* was predominant in spring varieties released in the United States and Argentina between 1970 and 2004 and hypothesized that the increase in *Vrn-A1a* frequency in this germplasm was related to the introduction of the semi-dwarf germplasm from CIMMYT during the 1970s. Indeed, the allele *Vrn-A1a* was not present in a collection of durum wheat landraces analyzed by Royo et al. [68], which were typically characterized by tall plants, long coleoptiles, and early vigor. Later, Muterko et al. [62] described three different variants of *Vrn-A1a* designated as *Vrn-A1a.1*, *Vrn-A1a.2*, and *Vrn-A1a.3*. *Vrn-A1a.1* and *Vrn-A1a.3* corresponded to the known *Vrn-A1a* allele described by Yan et al. [36] in hexaploid and tetraploid wheat, whereas *Vrn-A1a.2* was novel and compared to *Vrn-A1a* was characterized by two deletions (16 bp and 4 bp) within the MITE element [36,37,62] (Figure 1, Figure 2 and Figure 3; Table S1).

Yan et al. [36] also described the allele called *Vrn-A1b* in tetraploid accession (*T. durum*), which is characterized by a 20 bp deletion in the 5-UTR and two mutations in the identical host direct duplications (HDD) region. Within the *Vrn-A1b* allele, six variants (*Vrn-A1b.1*, *Vrn-A1b.2*, *vrn-A1b.3*, *vrn-A1b.4*, *Vrn-A1b.5*, and *Vrn-A1b.6*) were described by Muterko et al. [62]. These alleles are characterized by variants within the A-tract and C-rich regions of the VRN-box [62,69]. Mutations within the VRN-box differentiate variants of *Vrn-A1b* and *Vrn-A1i* from the recessive *vrn-A1* [62]. The VRN-box of all *Vrn-A1b* alleles along with *Vrn-A1i* harbor polymorphisms in the A-tract, although *Vrn-A1b.5* and *Vrn-A1b.6* can be distinguished from *Vrn-A1i* and *vrn-A1b.4* by an SNP within the C-rich segment. Moreover, Konopatskaia et al. [64] described a novel allele, named *Vrn-A1b.7*, characterized by deletions of 20 bp located 137 bp upstream of the start codon and mutations within the VRN-box (Figure 1, Figure 2 and Figure 3; Table S1).

Tranquilli and Dubcovsky [70] also identified variants within the VRN-box. *vrn-A^m^1* and *Vrn-A^m^2* were found in diploid *T. monococcum* and were reported as dominant for spring and winter growth habits. Sequence analysis revealed SNPs in the A-tract of the VRN-box in *T. turgidum* and *T. durum* and validated the identification of the *vrn-A^m^1* allele for the accession of *T. monococcum* [69]. Subsequently, Muterko et al. [69] demonstrated the existence of a 10 bp deletion in diploid wheat (*T. monococcum*), as well as some natural variants, *Vrn-A^m^1a*, *vrn-A^m^1b* [67], and *Vrn-A^m^1g* [20], that exhibited deletions or a complete absence of the CArG-box, as in the specific case of *vrn-A^m^1b*. Natural variants within the other regulatory regions (i.e., CarG box and/or G box) were also identified in tetraploid and hexaploid wheat [20,62,71]. Two alleles, named *Vrn-A1d* and *Vrn-A1e*, harbored 32 bp and 54 bp deletions within the CarG box, respectively [36], whereas *Vrn-A1f* exhibited a substantial 50 bp deletion within the −62 and −112 bp region; it also displayed a smaller 8 bp deletion within the G box [71] as well as a polymorphism within the A-tract (A replaced by G) [69]. This allele was first described by Golovnina et al. [71] in a collection of wild diploids (*T. boeoticum* and *T. urartu*) and tetraploid (*T. araraticum* and *T. timopheevii*) wheat. In addition to *Vrn-A1f*, Golovnina et al. [71] described two other variants called *Vrn-A1g* and *Vrn-A1h* as having 34 bp and 20 bp deletions near the CArG- box, respectively, in addition to the minor deletion of 8 bp in the G box. Among them, the dominant *Vrn-A1g* allele was reported as extremely rare in both *T. monococcum* and *T. boeoticum* [71]. Ivaničová et al. [72] designed a *Vrn-A1f-like* allele from *T. militinae* (Zhuk. and Migush.) (2n = 4x = 28, AtGG genome), a wild wheat that originated from a hybridization event separate from emmer wheat and belongs to the *T. timopheevii* (Zhuk.) group. Comparison between *Vrn-A1f-like* and *Vrn-A1a* revealed major mutations in the promoter region [the nonexistence of the Spring fold element (SFE) insertion and two deletions (8 base pairs and 50 base pairs) positioned downstream of the CArG box] but also within the first intron [72]. In spring *T. dicoccum*, a dominant allele known as *Vrn-A1k*, characterized by a 42 bp insertion at −108 bp, was reported by Muterko and Salina [73], whereas *Vrn-A1j* was described in *T. compactum* as carrying a deletion of 54 bp between −140 and −87 in the promoter [74] (Figure 1; Table S1).

Miroshnichenko et al. [75] reported the absence of overlapping between *Vrn-A1f*, *Vrn-A1k*, and *Vrn-A1j* and the majority of known VRN box-related mutations. Recently, Zhang et al. [76] discovered two additional alleles (*vrn-A1n* and *Vrn-A1o*) that showed a linked duplication in the promoter region. The common copy was identical to the recessive allele *vrn-A1*, whereas the additional copy of *vrn-A1n* contained a 54 bp deletion in the promoter that did not influence flowering, and *Vrn-A1o* harbored an 11 bp deletion that conferred spring growth [76] (Figure 1; Table S1).

Regarding the *Vrn-B1* gene, Chu and colleagues [22] revealed a novel retrotransposon located at ~100 bp upstream of the start codon at the promoter level of the *Vrn-B1* allele. This retrotransposon is responsible for conferring the spring growth habit in durum wheat [22]. Later, Muterko et al. [69] recognized novel allelic variants in the promoter regions of *Vrn-B1* by investigating 178 tetraploid and hexaploid wheat accessions. The new variants were named *Vrn-B1.f*, *Vrn-B1.s*, and *Vrn-B1.m* for *Vrn-B1* and were extensively distributed in both hexaploid and tetraploid wheat. Furthermore, the novel allele *Vrn-B1.m*, along with *Vrn-B1.m*.1 and *Vrn-B1.m*.2, was detected by Muterko and collaborators [62] in *T. dicoccum*, and characterized by a polymorphism and deletion in the promoter region (Figure 1; Table S1).

As for *Vrn-D1*, compared with the recessive *vrn-D1*, the allele named *Vrn-D1c* harbors a 174 bp insertion in bread wheat [77,78] (Figure 1; Table S1).

Finally, novel allele *Vrn-G1* and *Vrn-G1.a* were detected by Shcherban and collaborators [79] in *T. timopheevii*, characterized by an insertion of 215 bp at −99 bp in the promoter region (Figure 1; Table S1).

### 3.2. Allelic Variation of Vrn-1 at Gene Body Level

Regarding the allelic variation at the gene body level, *Vrn-A1c* [37] and *Vrn-A1L* [80] alleles were discovered in tetraploid wheat, which were characterized by 5.5 kb and 7.2 kb deletions in the first intron, respectively [80] (Figure 1, Figure 2 and Figure 3; Table S1). Compared to the recessive *vrn-A1* allele, *Vrn-A1c* in hexaploid wheat had eight unique SNPs and five unique 1 bp indels in the first intron [37]. Additionally, an allele called *Vrn-A1ins* was identified, which possesses a 0.5 kb insertion within intron 1 of the diploid *T. monococcum* [80]. Furthermore, the *vrn-A1u* allele was observed, and was characterized by a 1.4 kb deletion within intron 1 of *T. urartu* and polyploid species with an A-genome [80]. Sehgal et al. [81] and Steinfort et al. [82] described the *Vrn-A1f* and *VRN-A1AUS28709 Ai2* alleles in *T. aestivum*, respectively, harboring a deletion in intron 1. Furthermore, *T. araraticum* and *T. timopheevii* as the tetraploid species of the *Timopheevi* group are characterized by *Vrn-A1f-del* (2.7 kb deletion at intron 1 in *T. araraticum), Vrn-A1f-ins* (0.4 kb insertion at intron 1 in *T. timopheevii*), and *Vrn-A1f-del/ins* (0.4 kb insertion and 2.7 kb deletion at intron 1 in *T. timopheevii*), plus the deletions and the polymorphism in the promoter as described for allele *Vrn-A1f* [79], while *T. militinae* possesses an MITE transposon (0.4 kb insertion) and a 2.7 kb deletion in intron 1, and also exhibits a host duplication of nine base pairs in the first intron, and two synonymous SNPs in exon 7 and exon 8 [72]. Intriguingly, a polymorphism in the coding sequence of the recessive allele has been exclusively identified for *Vrn-A1* [83,84]. Based on the presence of “C → T” transition within exon 4 at position 20 bp of *Vrn-A1*, two different haplotypes were initially distinguished (Ex4C, wild type and Ex4T, mutant type). Similarly, the same transition (“C → T”) which led to the substitution of alanine for valine (Ala180/Val180) within exon 7 was observed [83]. Muterko and Salina [74] reported then a survey of exon 4 haplotypes in 12 tetraploid and hexaploid wheat species. The authors found that the Ex4T haplotype was present only in the hexaploid wheat *vrn-A1* allele, and exclusively in combination with the Ex4C haplotype in accessions of hexaploid wheat carrying *Vrn-A1* multi-copies. In addition, to denote the *Vrn-A1* exon 4 haplotype, Muterko and Salina used the previously available nomenclature [73], further expanding it. Using the abovementioned nomenclature, mutations within intron-4 were used to distinguish four haplotypes (Ex4C.s, Ex4C.m, Ex4C.f, and Ex4C.sph) [73]. The first three were named based on their migration velocity (s: slow, m: middle, f: fast), whereas Ex4C.sph was detected only in *T. sphaerococcum*. Furthermore, Muterko and Salina [74] identified two polymorphisms in exon 4 and exon 7 on the *Vrn-A1j* (exon 7) and *Vrn-A1k* (both exon 4 and 7) alleles.

The dominant alleles of the *Vrn-B1* and *Vrn-D1* loci exhibit variations from the recessive alleles, mainly characterized by insertions or deletions within the first intron [37,56,85]. The allele *Vrn-B1a*, identified in 2005 by Fu and colleagues [37], was characterized by a 6850 bp deletion in intron 1, whereas a similar allele called *Vrn-B1b* (the same 6850 bp deletion of *Vrn-B1a* plus a 36 bp indel) was described by Santra et al. [86]. *Vrn-B1c*, discovered by Chu et al. [22] and later by Milec et al. [87], differs from the others by an 817 bp deletion and 432 bp duplication in intron 1. Zhang et al. [88] reported a novel dominant allele, *Vrn-B1d*, in the Chinese spring Hongchunmai. The allele contained several genetic divergences within intron 1 compared to *vrn-B1*, including a large 6850 bp deletion (670–7519 bp), one small 187 bp deletion (7851–8037 bp), an SNP (T/C at 7845 bp), and one 4 bp mutation (TTTT to ACAA, 7847–7850 bp). In 2021, Strejčková and colleagues [63] found a novel allele called *Vrn-B1f*, which was characterized by an 836 bp insertion within intron 1 in bread wheat.

Recently, several mutations in both encoding and non-encoding regions of *Vrn-A1* and *Vrn-B1* were identified in 95 out of 263 wild emmer (*T. dicoccoides*) wheat genotypes with diverse growth habits and flowering times collected in the Fertile Crescent [89]. In 2023, Strejčková et al. [89] identified 15 and 7 SNPs in exons of *Vrn-A1* and *Vrn-B1*, respectively, as well as one insertion in exons of *Vrn-B1*. The *Vrn-D1a* and *Vrn-D1b* dominant alleles of spring bread wheat have the same deletion (4235 bp) but they differ from each other by an SNP (C/A at translation site of CArG-box in *Vrn-D1b*) [37,90].

### 3.3. Copy Number Variations of Vrn-1

Copy number variation (CNV) can also greatly impact *Vrn-1* gene function [65], thus influencing wheat adaptation and flowering time [65,91,92]. In bread wheat, CNV in recessive and dominant *Vrn-1* alleles has been reported [65,92,93]. A different number of copies of *Vrn-A1* led to different vernalization requirements among winter wheat cultivars [65,91]. The heading date of winter wheat was affected by allelic variation associated with CNV at the *Vrn-A1* locus [94]. The earlier flowering after a short vernalization period relates to a low copy number at *Vrn-A1* [65]. In other words, the CNV of the *Vrn-A1* gene strongly impacts vernalization requirements and late flowering [65]. Zhu et al. [91] recommended that choosing wheat varieties with three copies of the recessive *vrn-A1* gene would be a viable method to increase the frost tolerance ability of wheat because of the association between increased *Vrn-A1* copy number and greater frost tolerance.

More than 90% of winter varieties of *T. aestivum* carry two to three copies of the *Vrn-A1* gene [92]. Muterko and Salina [93] represented the copy number of *Vrn-A1* with the alternative exon 4 haplotype in spring and winter accessions of tetraploid and hexaploid wheat. Another study reported the duplication of *Vrn-A1b.3* in *T. dicoccum* and the *Vrn-A1b.3* and *Vrn-A1b.2* in hexaploid *T. spelta* [95]. Muterko [95] described that duplicated *Vrn-A1b.2* was related to the awnless spikes in *T. spelta*, whereas Würschum et al. [92] found that the geographical patterns of *Vrn-A1* copy number variations were compatible with their roles in promoting wheat’s worldwide adaptability.

CNV at the *Vrn-B1* locus was also reported by Muterko and Salina [93] in *T. compactum* (Host) and *T. spelta* (L.), although Strejčková et al. [63] reported that *Vrn-B1* and *Vrn-D1* exist in a single copy. By contrast, the authors found that recessive *Vrn-A1* has one to four copies, whereas the dominant *Vrn-A1* has one or two copies [63].

### 3.4. Allelic Variation of Vrn-1 at Different Ploidy Levels

**Figure 4:**
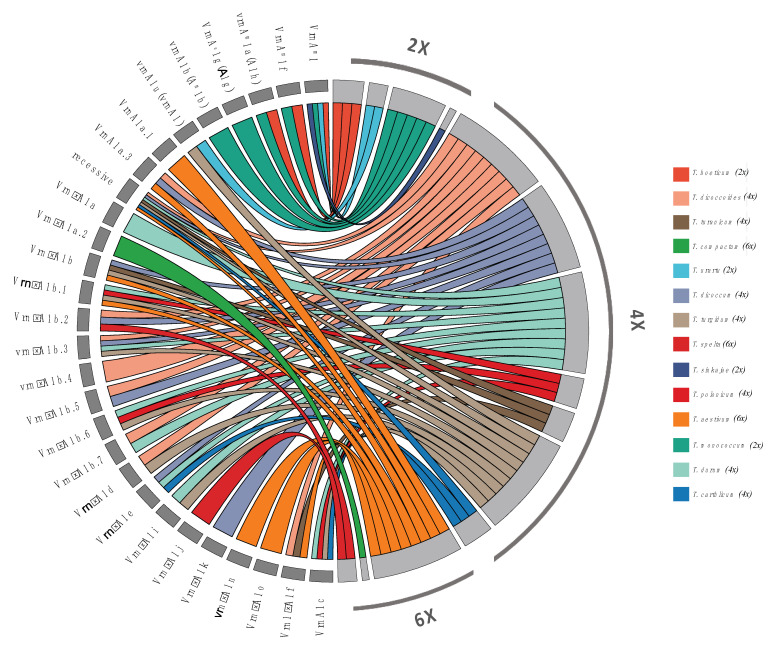
Chord diagram illustrating the allelic variation of *Vrn-1*. Colors represent the allelic variations, changing based on their association with different wheat as well as ploidy groups: diploid (2x), tetraploid (4x), and hexaploid (6x). The plot was drawn using the chordDiagram() implemented in the R library circlize (version 0.4.15) [97].

On the AA genome, three recessive alleles (*vrn-A^m^1*, *vrn-A1u*, and *vrn-A^m^1b*) have been identified in diploid species [19,20,71,80] (Figure 4).

The *vrn-A^m^1* allele was found in all diploid species, and to date, it represents the only variant reported in *Triticum sinskajae* A. Filat. et Kurk. [19,20,71,80]. By contrast, *vrn-A1u*, identified in *T. urartu* Thum. ex Gandil by Golovnina et al. [71], is identical to the recessive *vrn-A1* reported in polyploid wheat and differs from *vrn-A^m^1* for a deletion in the promoter region [71,80]. The *vrn-A^m^1b* allele instead was only detected in accessions of *T. monococcum* L. [19,67]. Dominant alleles were also identified in diploid wheat (e.g., *T. monococcum*) [71]. For example, two dominant alleles (*Vrn-A^m^1f* and *Vrn-A^m^1a Vrn-A1h*) were found in *T. boeoticum* Boiss. and *T. monococcum* [71,80], whereas, so far, no dominant alleles have been identified in *T. urartu* [71,80].

In tetraploid species, the recessive allele *vrn-A1* was inherited from diploids, presumably from *T. urartu,* since no differences were observed at the promoter level (Figure 3) [64,80], and to date, three recessive alleles [*vrn-A1*(*vrn-A1u*), *vrn-A1b.3*, *vrn-A1b.4*] have been described in both *Timopheevii* A. Filat. et Dorof. and *Dicoccoides* Flaksb. sections [62,69,80]. As suggested by Konopatskaia et al. [64], dominant alleles such as *Vrn-A1a.3*, *Vrn-A1e*, *Vrn-A1i* and *Vrn-A1b* might originate through deletion (*Vrn-A1b* and *Vrn-A1e*), insertion (*Vrn-A1a.3*), or substitution (*Vrn-A1i*) events from the recessive *vrn-A1.* Interestingly, dominant alleles of *Vrn-A1b* except *Vrn-A1b.7* and *Vrn-A1e* were distributed only in the dicoccoides section (AABB), suggesting that they evolved from *vrn-A1* after the section separation [34,64]. By contrast, *Vrn-A1b.7* was found in both the Emmer lines (AABB) and the *Timopheevii* lines (AAGG), suggesting that they originated from a common tetraploid ancestor [64]. Shcherban and Salina [85] reported that the presence of new dominant *Vrn-1* alleles was not related to the origin in diploids, since the allele set found in *T. dicoccoides* differs from *Timopheevii,* indicating an independent origin of dominant alleles within these two allopolyploids [85]. In *T. timopheevii* Zhuk. and *T. araraticum* Jakubz. have only one dominant allele (*Vrn-A1f*), which originated from the recessive *vrn-A^m^1* of *T. monococcum*, *T. urartu*, *T. boeoticum*, and was described at the *Vrn-A1* locus [32], whereas ten dominant alleles were identified in different tetraploid wheat species of section *Dicoccoides* Flaksb. [*Vrn-A1a*(*Vrn-A1a.3*), *Vrn-A1b*(*Vrn-A1b.1*), *Vrn-A1b.2*, *Vrn-A1b.5*, *Vrn-A1b.6*, *Vrn-A1e*, *Vrn-A1f*, *Vrn-A1i*, and *Vrn-A1d*] [34,69,71,80]. *Vrn-A1a.3* was restricted to *T. dicoccum* and *T. dicoccoides,* whereas the dominant *Vrn-A1d* allele has been found in both *Timopheevii* A. Filat. et Dorof. and *Dicoccoides* Flaksb. sections and it probably arises from *Vrn-A1b* variants due to an extended deletion. Konopatskaia et al. [64] alternatively reported that the two deletions within *vrn-A1* could originate from the *Vrn-A1d* locus [64]. *Vrn-A1d* probably originated at the tetraploid level, and it was not inherited in hexaploid wheat, as suggested by Konopatskaia et al. [64], even though most of the known dominant *Vrn-1* alleles in common hexaploid wheat originated at the tetraploid stage [*Vrn-A1a.1*, *Vrn-A1a.2*, *Vrn-A1b*(*Vrn-A1b.1*), *Vrn-A1b.2*, *Vrn-A1b.6*, *Vrn-A1c*, and *Vrn-A1f*] [22,37,77,96].

In hexaploid wheat, before the identification of *vrn-A1b.3* in *T. vavilovii* (Thum.) Jakubz. and *T. spelta* L. by Muterko et al. [62,69], *vrn-A1* was the only recessive allele identified [36,37,62].

In tetraploid wheat, four dominant alleles at the *Vrn-B1* locus were described [62,69], each characterized by mutations within the promoter region (such as insertion of repeated elements or short deletions) [22,69,71,98].

*Vrn-B1a* is the only dominant allele identified in the *dicoccoides* section and *durum* accessions [37,69,71], whereas *Vrn-B1c* probably originated from *Vrn-B1a* due to an additional deletion of 0.8 kb and a duplication of 0.4 kb [62]. Also, the *Vrn-B1b* allele appears to have originated from *Vrn-B1a*, since along with a deletion in the first intron, it also harbors a 36 bp deletion plus an additional SNP [86]. This allele was described in common wheat originating from North America and was associated with the spring growth habit [96]. The *Vrn-B1dic* promoter differs from *vrn-B1* for 29 nucleotide substitutions, one deletion, and one SNP insertion in the region spanning −220 to −155 bp upstream of the start codon, and it was found only in a genotype belonging to *T. dicoccoides* [64].

Shcherban et al. [85] identified one accession of *T. turanicum* Jakubz. (AABB) with the *Vrn-B1a* allele that does not correspond to the dominant *Vrn-B1a* for an insertion in the promoter [71]. Interestingly, the insertion was homologous to that identified in the *Vrn-A1a* allele, although the position was different (−100 from the start codon).

The dominant *Vrn-D1a* allele was found in the near-isogenic line TDE and it abounded in spring wheat adapted to tropical and subtropical regions [99,100]. *Vrn-D1b* arises from *Vrn-D1a* due to SNP in the CArG-box region [90]. The *Vrn-D1c* allele was found in three out of 205 Chinese wheat cultivars [77]. In the same year, Muterko et al. [101] found the *Vrn-D1s* allele, which is associated with spring form. Shcherban et al. [85] reported that the distribution of spring forms along with different alleles at *Vrn-1* is largely due to artificial selection based on different climatic conditions. For example, dominant haplotypes at the *Vrn-A1* and *Vrn-B1* loci were observed in cultivars from northern and central Europe and from Russia [85], whereas the monogenic dominant haplotypes contained at either *Vrn-B1* or *Vrn-D1* were mostly widespread in cultivars for southern Europe [85,102]. Therefore, the monogenic *Vrn-B1*/*Vrn-D1* haplotypes were suggested to gain a breeding advantage for subtropical southern regions, providing a longer vegetative period [85]. By contrast, digenic dominant haplotypes might be useful in regions with a temperate climate [103].

Unfortunately, previous studies focused only on the promoter or gene body levels, whereas most of the other studies covered accessions of a single species or a specific ploidy level. *Vrn-A1f* is a good example. Golovnina et al. [71] described the *Vrn-A1f* allele at the promoter level in a collection of four diploid wheat species (*T. urartu*, *T. boeoticum*, *T. monococcum*, and *T. sinskajae*), seven genotypes belonging to *Aegilops speltoides* and *Ae. squarrosa* (syn. *Ae. tauschii*), and 17 accessions belonging to the sections of *Dicoccoides*, *Triticum*, *Timopheevii*. Later, Sehgal et al. [81] described a 6Kb deletion in intron 1, naming the allele as *Vrn-A1f* in wheat germplasm composed of landrace accessions, synthetic hexaploids developed at CIMMYT by crossing durum wheat (*T. turgidum* subsp. *durum*) or emmer wheat (*T. turgidum* subsp. *dicoccum*) with diverse *Aegilops tauschii* accessions, and elite lines. However, the promoter region was not covered and investigated, thus leaving gaps at both species and gene levels. Similarly, the *Vrn-B1c* allele reported by Milec et al. [104] and Chu et al. [22] was renamed as *Vrn-B1d* in the Catalog of Gene Symbols for Wheat [105], but it is unrelated to the *Vrn-B1d* allele reported by Zhang et al. [88]. In addition, in most cases, the focus was carried out without considering the CNV at the *Vrn-A1* gene. For example, roughly 90% of winter hexaploid wheat varieties carry two to three copies of this gene [92], with different haplotypes at exon 4 and exon 7 [65,106,107].

### 3.5. Allelic Variation of Vrn-2, Vrn3, and Vrn4 Genes

The identification of natural variations in *Vrn-2* genes may prove difficult due to the limited characterization of the *Vrn-2* gene in hexaploid wheat. Indeed, few natural variations in the promoter and/or in the first intron of *Vrn-2* genes (*Vrn-A2*, *Vrn-B2*, *Vrn-D2*, and *Vrn-S2*) were identified and characterized (Figure 1; Table S2). They were originally observed in diploid wheat (*T. monococcum*) [25]. Furthermore, a previous development of a tetraploid wheat line lacking functional copies of *Vrn-2* has been documented [104]. In addition, various hexaploid wheat cultivars may have undergone multiple events of duplication, deletion, and translocation involving *Vrn-B2*. Consequently, the task of identifying specific variations becomes challenging [39]. Unlike *Vrn-1*, *Vrn-3*, and *Vrn-4* genes that are dominant for spring growth habit, *Vrn-2* genes are dominant for winter growth habit [25]. *Vrn-B2* is generally functional, whereas *Vrn-A2* is non-functional in tetraploid wheat [40,108]. Tan and Yan [39] isolated *Vrn*-*2* from hexaploid winter wheat cultivars Jagger and 2174, reporting no differences at *Vrn*-*A2* or *Vrn*-*D2*, while two copies of *Vrn*-*B2* were found in 2174, indicating that Jagger carried a *null* allele. The first copy (*Vrn*-*B2a*.*1*) was 2327 bp long and had a 2087 bp insertion between the start and stop codon plus a 144 bp insertion before the start codon, and a 96 bp insertion after the stop codon, whereas *Vrn*-*B2a.2* had an extra ‘CAC’ motif at positions 136–138 from the start codon and five SNPs compared with *Vrn*-*B2a.1* [39]. The cloned *Vrn-D2* was 2364 bp in length, where 239 bp corresponded to an insertion before the start codon and 96 bp to an insertion after the stop codon [39]. Distelfeld et al. [108] reported *Vrn-S2* in *Ae. speltoides* and *Vrn-D2* in *Ae. tauschii,* concluding that the winter growth habit of most of the *Ae. speltoides* and *Ae. tauschii* accessions was probably due to functional *Vrn-2*. The ZCCT1 and ZCCT2 proteins from both species showed no mutations in the conserved amino acids of the CCT domains [108].

Several natural variations were also detected and characterized in the promoter and/or in the first intron of *Vrn-3* (*Vrn-A3*, *Vrn-B3*, and *Vrn-D3*) (Figure 1; Table S3). Recently, Nishimura et al. [109] reported in wild emmer wheat six *Vrn-A3* alleles with the 7- and 25 bp insertions in the promoter region, namely, *Vrn-A3a-h2*, *Vrn-A3a-h3*, *Vrn-A3a-h4*, *Vrn-A3a-h5*, *Vrn-A3a-h6*, and *Vrn-A3c-h2*. Similar insertions (i.e., *Vrn-A3a-h2* and *Vrn-A3c-h1*) were also found in cultivated tetraploid and hexaploid wheat [109]. Yan et al. [26] identified the *vrn-A^m^3* allele in *T. monococcum*, which is characterized by a polymorphism in the promoter region. The *Vrn-B3* locus in tetraploid and hexaploid wheat is defined by five dominant alleles, all linked to modifications in the promoter. Yan et al. [26] identified the *Vrn-B3a* allele characterized by the insertion of 5300 bp in the promoter region. Later, Chen et al. [84] showed two novel alleles: *Vrn-B3b*, with an insertion of 890 bp in the promoter, and *Vrn-B3c*, characterized by two deletions (20 bp and a 4 bp) in the promoter of *Vrn-B3a*. Berezhnaya et al. [110] discovered two novel allelic variants of the *Vrn-B3* gene in common wheat varieties from Russia. These alleles were designated the *Vrn-B3d* and *Vrn-B3e* alleles and had 1615 bp and 160 bp insertions in the promoter, respectively [110]. Among the alleles described for *Vrn-3*, Muterko et al. [62] reported a high frequency of *Vrn-B3a* in *T. durum* varieties from Ukraine, Russia, and Kazakhstan. Finally, Bonnin et al. [42] demonstrated the presence of polymorphic sites within four haplotypes in the A genome (*TAFTAh1*, *TAFTAh2*, *TaFTAh3*, and *TAFTAh4*), whereas two were identified in the D genome (*TAFTDh1* and *TAFTDh2*), and only one line (BT21) showed a polymorphism in the B genome (*TaFTBBT21*) of *Vrn-3*. All five affected sites (three SNPs and two deletions) were found within the first intron [43]. Additionally, a single polymorphism for genome D was observed, consisting of an INDEL of one G in the third exon [43].

*Vrn-4* is an early flowering allele and is comparatively less comprehended in comparison to the preceding three vernalization genes. The Australian cultivar Gabo was the first to identify *Vrn-4* [59,111], and subsequently, it was backcrossed into Triple Dirk to produce an isogenic line called TDF [111]. This locus was assigned to chromosome 5D [112] and is now recognized as *Vrn-D4* [113]. Although only the D genome has been identified thus far as having the natural variation for flowering time in the centromeric region of homologous group 5 chromosomes, the arm position of *Vrn-D4* in wheat is yet unclear [44]. The *Vrn-D4* locus might play a crucial role in the variation in flowering time in hexaploid wheat germplasm, and it seems to have undergone independent evolution from the vernalization pathway in dicot species [25].

## 4. Conclusions and Future Perspectives

Phenology is a key target for adapting wheat varieties to ongoing climate changes and *Vrn* genes play a crucial role in accelerating reproductive development after prolonged exposure to low temperatures during the winter [85,114,115,116,117]. Matching the development phases and growth of the wheat crop to the thermal regime of the growth environment allows us to maximize the exploitation of natural resources by limiting climatic stress.

This review highlights the effort of the scientific community to understand the molecular mechanisms underlying wheat vernalization in the last two decades. Molecular advances have not only unveiled a rich reservoir of allelic variability within *Vrn-1* genes, impacting critical developmental stages, but they have also brought to light the complexity of the allelic landscape. However, the quest for a comprehensive understanding remains a captivating puzzle.

Recent advances in sequencing technologies can now help to untangle vernalization, covering all the gene space of *Vrn-1* in different species at one time. Precise genotyping of *Vrn-1* genes, by assigning weights to each *Vrn* allele, might not only improve the accuracy of genomic prediction models but also streamline the varietal selection process [118,119,120,121], identifying the best *Vrn* allelic combination. This information could also be used to feed phenological predictive models to rapidly identify genetic material with the optimum phenology within different target regions and/or untested advanced breeding lines early in a breeding program. Additionally, the possibility of inducing site-specific mutations in the genetic sequences of these genes offers new perspectives for developing resilient wheat varieties.

## Data Availability

Supporting materials are reported as Supplementary Tables: Tables S1–S3.

## Supplementary Materials

The following supporting information can be downloaded at: https://www.mdpi.com/article/10.3390/genes15020251/s1, Table S1. Survey of natural variations in *Vrn-1* genes: allele mutated, synonyms, GenBank accession code, year, ploidy, wheat genotypes, sequence variation, mutations, and reference are reported; Table S2. Survey of natural variations in *Vrn-2* genes: allele mutated, *ZCCT* genes, GenBank accession code, year, ploidy, wheat genotypes, sequence variation, mutation, and reference are reported. Table S3. Survey of natural variations in *Vrn-3* genes: allele mutated, note, GenBank accession code, year ploidy, wheat genotypes, sequence variation, mutation, and reference are reported.

## References

[1] Matsuoka Y. (2011). Evolution of Polyploid *Triticum* Wheats under Cultivation: The Role of Domestication, Natural Hybridization and Allopolyploid Speciation in Their Diversification. Plant Cell Physiol..

[2] Mourad A.M.I., Alomari D.Z., Alqudah A.M., Sallam A., Salem K.F.M. (2019). Recent Advances in Wheat (*Triticum* spp.) Breeding. Advances in Plant Breeding Strategies: Cereals.

[3] Sattler M.C., Carvalho C.R., Clarindo W.R. (2016). The Polyploidy and Its Key Role in Plant Breeding. Planta.

[4] Van De Peer Y., Mizrachi E., Marchal K. (2017). The Evolutionary Significance of Polyploidy. Nat. Rev. Genet..

[5] Van de Peer Y., Ashman T.L., Soltis P.S., Soltis D.E. (2021). Polyploidy: An Evolutionary and Ecological Force in Stressful Times. Plant Cell.

[6] Alix K., Gérard P.R., Schwarzacher T., Heslop-Harrison J.S.P. (2017). Polyploidy and Interspecific Hybridization: Partners for Adaptation, Speciation and Evolution in Plants. Ann. Bot..

[7] Dubcovsky J., Dvorak J. (2007). Genome Plasticity a Key Factor in the Success of Polyploid Wheat under Domestication. Science.

[8] Fang Z., Morrell P.L. (2016). Polyploidy Boosts Domestication. Nat. Plants.

[9] Taranto F., Esposito S., Fania F., Sica R., Marzario S., Logozzo G., Gioia T., De Vita P. (2023). Breeding Effects on Durum Wheat Traits Detected Using GWAS and Haplotype Block Analysis. Front. Plant Sci..

[10] Kilian B., Özkan H., Pozzi C., Salamini F., Muehlbauer G.J., Feuillet C. (2009). Domestication of the Triticeae in the Fertile Crescent BT—Genetics and Genomics of the Triticeae.

[11] Yang C., Zhao L., Zhang H., Yang Z., Wang H., Wen S., Zhang C., Rustgi S., Von Wettstein D., Liu B. (2014). Evolution of Physiological Responses to Salt Stress in Hexaploid Wheat. Proc. Natl. Acad. Sci. USA.

[12] Fowler D.B., Dvorak J., Gusta L. (1977). V Comparative Cold Hardiness of Several *Triticum* Species and *Secale cereale* L. Crop Sci..

[13] Limin A.E., Fowler D.B. (1981). Cold Hardiness of Some Relatives of Hexaploid Wheat. Can. J. Bot..

[14] Peng J., Sun D., Nevo E. (2011). Wild Emmer Wheat, “*Triticum dicoccoides*”, Occupies a Pivotal Position in Wheat Domestication Process. Aust. J. Crop Sci..

[15] Taranto F., Esposito S., De Vita P. (2023). Genomics for Yield and Yield Components in Durum Wheat. Plants.

[16] Distelfeld A., Li C., Dubcovsky J. (2009). Regulation of Flowering in Temperate Cereals. Curr. Opin. Plant Biol..

[17] Scarth R., Law C.N. (1983). The Location of the Photoperiod Gene, *Ppd2* and an Additional Genetic Factor for Ear-Emergence Time on Chromosome 2B of Wheat. Heredity.

[18] Law C.N., Worland A.J. (1997). Genetic Analysis of Some Flowering Time and Adaptive Traits in Wheat. New Phytol..

[19] Yan L., Loukoianov A., Tranquilli G., Helguera M., Fahima T., Dubcovsky J. (2003). Positional Cloning of the Wheat Vernalization Gene *VRN1*. Proc. Natl. Acad. Sci. USA.

[20] Dubcovsky J., Loukoianov A., Fu D., Valarik M., Sanchez A., Yan L. (2006). Effect of Photoperiod on the Regulation of Wheat Vernalization Genes *VRN1* and *VRN2*. Plant Mol. Biol..

[21] Stelmakh A.F. (1987). Growth Habit in Common Wheat (*Triticum aestivum* L. Em. Thell.). Euphytica.

[22] Chu C.G., Tan C.T., Yu G.T., Zhong S., Xu S.S., Yan L. (2011). A Novel Retrotransposon Inserted in the Dominant *Vrn-B1* Allele Confers Spring Growth Habit in Tetraploid Wheat (*Triticum turgidum* L.). G3 Genes Genomes Genet..

[23] Chhuneja P., Arora J.K., Kaur P., Kaur S., Singh K. (2015). Characterization of Wild Emmer Wheat *Triticum dicoccoides* Germplasm for Vernalization Alleles. J. Plant Biochem. Biotechnol..

[24] Boden S.A., McIntosh R.A., Uauy C., Krattinger S.G., Dubcovsky J., Rogers W.J., Xia X.C., Badaeva E.D., Bentley A.R., Brown-Guedira G. (2023). Updated Guidelines for Gene Nomenclature in Wheat. Theor. Appl. Genet..

[25] Yan L., Loukoianov A., Blechl A., Tranquilli G., Ramakrishna W., SanMiguel P., Bennetzen J.L., Echenique V., Dubcovsky J. (2004). The Wheat *VRN2* Gene Is a Flowering Repressor Down-Regulated by Vernalization. Science.

[26] Yan L., Fu D., Li C., Blechl A., Tranquilli G., Bonafede M., Sanchez A., Valarik M., Yasuda S., Dubcovsky J. (2006). The Wheat and Barley Vernalization Gene *VRN3* Is an Orthologue of FT. Proc. Natl. Acad. Sci. USA.

[27] Kippes N., Debernardi J.M., Vasquez-Gross H.A., Akpinar B.A., Budak H., Kato K., Chao S., Akhunov E., Dubcovsky J. (2015). Identification of the *VERNALIZATION 4* Gene Reveals the Origin of Spring Growth Habit in Ancient Wheats from South Asia. Proc. Natl. Acad. Sci. USA.

[28] Mandel M.A., Gustafson-brown C., Savidge B., Yanofsky M.F. (1992). Molecular Characterization of the *Arabidopsis* Floral Homeotic Gene *APETALA1*. Nature.

[29] Danyluk J., Kane N.A., Breton G., Limin A.E., Fowler D.B., Sarhan F. (2003). TaVRT-1, a Putative Transcription Factor Associated with Vegetative to Reproductive Transition in Cereals. Plant Physiol..

[30] Murai K., Miyamae M., Kato H., Takumi S., Ogihara Y. (2003). *WAP1*, a Wheat *APETALA1* Homolog, Plays a Central Role in the Phase Transition from Vegetative to Reproductive Growth. Plant Cell Physiol..

[31] Trevaskis B., Bagnall D.J., Ellis M.H., Peacock W.J., Dennis E.S. (2003). MADS Box Genes Control Vernalization-Induced Flowering in Cereals. Proc. Natl. Acad. Sci. USA.

[32] Pugsley A.T. (1971). A Genetic Analysis of the Spring-Winter Habit of Growth in Wheat. Aust. J. Agric. Res..

[33] Law C.N., Worland A.J., Giorgi B. (1976). The Genetic Control of Ear-Emergence Time by Chromosomes 5A and 5D of Wheat. Heredity.

[34] Worland A.J. (1996). The Influence of Flowering Time Genes on Environmental Adaptability in European Wheats. Euphytica.

[35] Barrett B., Bayram M., Kidwell K. (2002). Identifying AFLP and Microsatellite Markers for Vernalization Response Gene *Vrn-B1* in Hexaploid Wheat Using Reciprocal Mapping Populations. Plant Breed..

[36] Yan L., Helguera M., Kato K., Fukuyama S., Sherman J., Dubcovsky J. (2004). Allelic Variation at the *VRN-1* Promoter Region in Polyploid Wheat. Theor. Appl. Genet..

[37] Fu D., Szűcs P., Yan L., Helguera M., Skinner J.S., Von Zitzewitz J., Hayes P.M., Dubcovsky J. (2005). Large Deletions within the First Intron in *VRN-1* Are Associated with Spring Growth Habit in Barley and Wheat. Mol. Genet. Genom..

[38] Trevaskis B., Hemming M.N., Dennis E.S., Peacock W.J. (2007). The Molecular Basis of Vernalization-Induced Flowering in Cereals. Trends Plant Sci..

[39] Tan C.T., Yan L. (2016). Duplicated, Deleted and Translocated *VRN2* Genes in Hexaploid Wheat. Euphytica.

[40] Kippes N., Chen A., Zhang X., Lukaszewski A.J., Dubcovsky J. (2016). Development and Characterization of a Spring Hexaploid Wheat Line with No Functional *VRN2* Genes. Theor. Appl. Genet..

[41] Faure S., Higgins J., Turner A., Laurie D.A. (2007). The *FLOWERING LOCUS T-like* Gene Family in Barley (*Hordeum vulgare*). Genetics.

[42] Li C., Dubcovsky J. (2008). Wheat FT Protein Regulates *VRN1* Transcription through Interactions with FDL2. Plant J..

[43] Bonnin I., Rousset M., Madur D., Sourdille P., Dupuits C., Brunel D., Goldringer I. (2008). FT Genome A and D Polymorphisms Are Associated with the Variation of Earliness Components in Hexaploid Wheat. Theor. Appl. Genet..

[44] Yoshida T., Nishida H., Zhu J., Nitcher R., Distelfeld A., Akashi Y., Kato K., Dubcovsky J. (2010). *Vrn-D4* Is a Vernalization Gene Located on the Centromeric Region of Chromosome 5D in Hexaploid Wheat. Theor. Appl. Genet..

[45] Le Gouis J., Bordes J., Ravel C., Heumez E., Faure S., Praud S., Galic N., Remoué C., Balfourier F., Allard V. (2012). Genome-Wide Association Analysis to Identify Chromosomal Regions Determining Components of Earliness in Wheat. Theor. Appl. Genet..

[46] Wen M., Su J., Jiao C., Zhang X., Xu T., Wang T., Liu X., Wang Z., Sun L., Yuan C. (2022). Pleiotropic Effect of the Compactum Gene and Its Combined Effects with Other Loci for Spike and Grain-Related Traits in Wheat. Plants.

[47] Tamaki S., Matsuo S., Wong H.L., Yokoi S., Shimamoto K. (2007). Hd3a Protein Is a Mobile Flowering Signal in Rice. Science.

[48] Kardailsky I., Shukla V.K., Ahn J.H., Dagenais N., Christensen S.K., Nguyen J.T., Chory J., Harrison M.J., Weigel D. (1999). Activation Tagging of the Floral Inducer FT. Science.

[49] Kobayashi Y., Kaya H., Goto K., Iwabuchi M., Araki T. (1999). A Pair of Related Genes with Antagonistic Roles in Mediating Flowering Signals. Science.

[50] Yeung K., Seitz T., Li S., Janosch P., McFerran B., Kaiser C., Fee F., Katsanakis K.D., Rose D.W., Mischak H. (1999). Suppression of Raf-1 Kinase Activity and MAP Kinase Signalling by RKIP. Nature.

[51] Kroslak T., Koch T., Kahl E., Höllt V. (2001). Human Phosphatidylethanolamine-Binding Protein Facilitates Heterotrimeric G Protein-Dependent Signaling. J. Biol. Chem..

[52] Corbesier L., Vincent C., Jang S., Fornara F., Fan Q., Searle I., Giakountis A., Farrona S., Gissot L., Turnbull C. (2007). FT Protein Movement Contributes to Long-Distance Signaling in Floral Induction of *Arabidopsis*. Science.

[53] Turner A., Beales J., Faure S., Dunford R.P., Laurie D.A. (2005). Botany: The Pseudo-Response Regulator *Ppd-H1* Provides Adaptation to Photoperiod in Barley. Science.

[54] Kamran A., Iqbal M., Spaner D. (2014). Flowering Time in Wheat (*Triticum aestivum* L.): A Key Factor for Global Adaptability. Euphytica.

[55] Fu D., Dunbar M., Dubcovsky J. (2007). Wheat VIN3-like PHD Finger Genes Are up-Regulated by Vernalization. Mol. Genet. Genom..

[56] Shi C., Zhao L., Zhang X., Lv G., Pan Y., Chen F. (2019). Gene Regulatory Network and Abundant Genetic Variation Play Critical Roles in Heading Stage of Polyploidy Wheat. BMC Plant Biol..

[57] Iwaki K., Nakagawa K., Kuno H., Kato K. (2000). Ecogeographical Differentiation in East Asian Wheat, Revealed from the Geographical Variation of Growth Habit and *Vrn* Genotype. Euphytica.

[58] (2001). Iwaki; Haruna; Niwa; Kato Adaptation and Ecological Differentiation in Wheat with Special Reference to Geographical Variation of Growth Habit and *Vrn* Genotype. Plant Breed..

[59] Knott D.R. (1959). The Inheritance of Rust Resistance.: Iv. Monosomic Analysis of Rust Resistance and Some Other Characters in Six Varieties of Wheat Including Gabo and Kenya Farmer. Can. J. Plant Sci..

[60] O’Brien L., Morell M., Wrigley C., Appels R., Bonjean A.P., Angus W.J. (2001). Genetic Pool of Australian Wheats. The World Wheat Book.

[61] Sung S., Schmitz R.J., Amasino R.M. (2006). A PHD Finger Protein Involved in Both the Vernalization and Photoperiod Pathways in *Arabidopsis*. Genes Dev..

[62] Muterko A., Kalendar R., Salina E. (2016). Allelic Variation at the *VERNALIZATION-A1*, *VRN-B1*, *VRN-B3*, and *PHOTOPERIOD-A1* Genes in Cultivars of *Triticum durum* Desf. Planta.

[63] Strejčková B., Milec Z., Holušová K., Cápal P., Vojtková T., Čegan R., Šafář J. (2021). In-depth Sequence Analysis of Bread Wheat *Vrn1* Genes. Int. J. Mol. Sci..

[64] Konopatskaia I., Vavilova V., Kondratenko E.Y., Blinov A., Goncharov N.P. (2016). *VRN1* Genes Variability in Tetraploid Wheat Species with a Spring Growth Habit. BMC Plant Biol..

[65] Díaz A., Zikhali M., Turner A.S., Isaac P., Laurie D.A. (2012). Copy Number Variation Affecting the *Photoperiod-B1* and *Vernalization-A1* Genes Is Associated with Altered Flowering Time in Wheat (*Triticum aestivum*). PLoS ONE.

[66] Kiseleva A.A., Salina E.A. (2018). Genetic Regulation of Common Wheat Heading Time. Russ. J. Genet..

[67] Pidal B., Yan L., Fu D., Zhang F., Tranquilli G., Dubcovsky J. (2009). The CArG-Box Located Upstream from the Transcriptional Start of Wheat Vernalization Gene *VRN1* Is Not Necessary for the Vernalization Response. J. Hered..

[68] Royo C., Dreisigacker S., Ammar K., Villegas D. (2020). Agronomic Performance of Durum Wheat Landraces and Modern Cultivars and Its Association with Genotypic Variation in Vernalization Response (*Vrn-1*) and Photoperiod Sensitivity (*Ppd-1*) Genes. Eur. J. Agron..

[69] Muterko A., Kalendar R., Salina E. (2016). Novel Alleles of the *VERNALIZATION1* Genes in Wheat Are Associated with Modulation of DNA Curvature and Flexibility in the Promoter Region. BMC Plant Biol..

[70] Tranquilli G., Dubcovsky J. (2000). Epistatic Interaction between Vernalization Genes *Vrn-Am1* and *Vrn-Am2* in Diploid Wheat. J. Hered..

[71] Golovnina K.A., Kondratenko E.Y., Blinov A.G., Goncharov N.P. (2010). Molecular Characterization of Vernalization Loci *VRN1* in Wild and Cultivated Wheats. BMC Plant Biol..

[72] Ivaničová Z., Jakobson I., Reis D., Šafář J., Milec Z., Abrouk M., Doležel J., Järve K., Valárik M. (2016). Characterization of New Allele Influencing Flowering Time in Bread Wheat Introgressed from *Triticum militinae*. New Biotechnol..

[73] Muterko A.F., Salina E.A. (2017). Analysis of the *VERNALIZATION-A1* Exon-4 Polymorphism in Polyploid Wheat. Vavilovskii Zhurnal Genet. I Sel..

[74] Muterko A., Salina E. (2018). Origin and Distribution of the *VRN-A1* Exon 4 and Exon 7 Haplotypes in Domesticated Wheat Species. Agronomy.

[75] Miroshnichenko D., Timerbaev V., Klementyeva A., Pushin A., Sidorova T., Litvinov D., Nazarova L., Shulga O., Divashuk M., Karlov G. (2022). CRISPR/Cas9-Induced Modification of the Conservative Promoter Region of *VRN-A1* Alters the Heading Time of Hexaploid Bread Wheat. Front. Plant Sci..

[76] Zhang B., Guo Y., Fan Q., Li R., Chen D., Zhang X. (2022). Characterization and Distribution of Novel Alleles of the Vernalization Gene *Vrn-A1* in Chinese Wheat (*Triticum aestivum* L.) Cultivars. Crop J..

[77] Zhang X., Gao M., Wang S., Chen F., Cui D. (2015). Allelic Variation at the Vernalization and Photoperiod Sensitivity Loci in Chinese Winter Wheat Cultivars (*Triticum aestivum* L.). Front. Plant Sci..

[78] Chepurnov G.Y., Ovchinnikova E.S., Blinov A.G., Chikida N.N., Belousova M.K., Goncharov N.P. (2023). Analysis of the Structural Organization and Expression of the *Vrn-D1* Gene Controlling Growth Habit (Spring vs. Winter) in *Aegilops taushii* Coss. Plants.

[79] Shcherban A.B., Schichkina A.A., Salina E.A. (2016). The Occurrence of Spring Forms in Tetraploid Timopheevi Wheat Is Associated with Variation in the First Intron of the *VRN-A1* Gene. BMC Plant Biol..

[80] Shcherban A.B., Strygina K.V., Salina E.A. (2015). *VRN-1* Gene- Associated Prerequisites of Spring Growth Habit in Wild Tetraploid Wheat *T. dicoccoides* and the Diploid A Genome Species. BMC Plant Biol..

[81] Sehgal D., Vikram P., Sansaloni C.P., Ortiz C., Pierre C.S., Payne T., Ellis M., Amri A., Petroli C.D., Wenzl P. (2015). Exploring and Mobilizing the Gene Bank Biodiversity for Wheat Improvement. PLoS ONE.

[82] Steinfort U., Trevaskis B., Fukai S., Bell K.L., Dreccer M.F. (2017). Vernalisation and Photoperiod Sensitivity in Wheat: Impact on Canopy Development and Yield Components. Field Crops Res..

[83] Sherman J.D., Yan L., Talbert L., Dubcovsky J. (2004). A PCR Marker for Growth Habit in Common Wheat Based on Allelic Variation at the *VRN-A1* Gene. Crop Sci..

[84] Chen F., Gao M., Zhang J., Zuo A., Shang X., Cui D. (2013). Molecular Characterization of Vernalization and Response Genes in Bread Wheat from the Yellow and Huai Valley of China. BMC Plant Biol..

[85] Shcherban A.B., Salina E.A. (2017). Evolution of *VRN-1* Homoeologous Loci in Allopolyploids of *Triticum* and Their Diploid Precursors. BMC Plant Biol..

[86] Santra D.K., Santra M., Allan R.E., Campbell K.G., Kidwell K.K. (2009). Genetic and Molecular Characterization of Vernalization Genes *Vrn-A1*, *Vrn-B1*, and *Vrn-D1* in Spring Wheat Germplasm from the Pacific Northwest Region of the U.S.A. Plant Breed..

[87] Milec Z., Tomková L., Sumíková T., Pánková K. (2012). A New Multiplex PCR Test for the Determination of *Vrn-B1* Alleles in Bread Wheat (*Triticum aestivum* L.). Mol. Breed..

[88] Zhang B., Wang X., Wang X., Ma L., Wang Z., Zhang X. (2018). Molecular Characterization of a Novel Vernalization Allele *Vrn-B1d* and Its Effect on Heading Time in Chinese Wheat (*Triticum aestivum* L.) Landrace Hongchunmai. Mol. Breed..

[89] Strejčková B., Mazzucotelli E., Čegan R., Milec Z., Brus J., Çakır E., Mastrangelo A.M., Özkan H., Šafář J. (2023). Wild Emmer Wheat, the Progenitor of Modern Bread Wheat, Exhibits Great Diversity in the *VERNALIZATION1* Gene. Front. Plant Sci..

[90] Zhang J., Wang Y., Wu S., Yang J., Liu H., Zhou Y. (2012). A Single Nucleotide Polymorphism at the *Vrn-D1* Promoter Region in Common Wheat Is Associated with Vernalization Response. Theor. Appl. Genet..

[91] Zhu J., Pearce S., Burke A., See D.R., Skinner D.Z., Dubcovsky J., Garland-Campbell K. (2014). Copy Number and Haplotype Variation at the *VRN-A1* and Central FR-A2 Loci Are Associated with Frost Tolerance in Hexaploid Wheat. Theor. Appl. Genet..

[92] Würschum T., Boeven P.H.G., Langer S.M., Longin C.F.H., Leiser W.L. (2015). Multiply to Conquer: Copy Number Variations at *Ppd-B1* and *Vrn-A1* Facilitate Global Adaptation in Wheat. BMC Genet..

[93] Muterko A., Salina E. (2019). *VRN1*-Ratio Test for Polyploid Wheat. Planta.

[94] Grogan S.M., Brown-Guedira G., Haley S.D., McMaster G.S., Reid S.D., Smith J., Byrne P.F. (2016). Allelic Variation in Developmental Genes and Effects on Winter Wheat Heading Date in the U.S. Great Plains. PLoS ONE.

[95] Muterko A. (2019). Copy Number Variation of the *Vrn-A1b* Allele in Emmer and Spelt Wheat. Curr. Chall. Plant Genet. Genom. Bioinform. Biotechnol..

[96] Milec Z., Sumíková T., Tomková L., Pánková K. (2013). Distribution of Different *Vrn-B1* Alleles in Hexaploid Spring Wheat Germplasm. Euphytica.

[97] Gu Z., Gu L., Eils R., Schlesner M., Brors B. (2014). Circlize implements and enhances circular visualization in R. Bioinformatics.

[98] Shcherban A.B., Efremova T.T., Salina E.A. (2012). Identification of a New *Vrn-B1* Allele Using Two near-Isogenic Wheat Lines with Difference in Heading Time. Mol. Breed..

[99] Zhang X.K., Xiao Y.G., Zhang Y., Xia X.C., Dubcovsky J., He Z.H. (2008). Allelic Variation at the Vernalization Genes *Vrn-A1*, *Vrn-B1*, *Vrn-D1*, and *Vrn-B3* in Chinese Wheat Cultivars and Their Association with Growth Habit. Crop Sci..

[100] Eagles H.A., Cane K., Kuchel H., Hollamby G.J., Vallance N., Eastwood R.F., Gororo N.N., Martin P.J. (2010). Photoperiod and Vernalization Gene Effects in Southern Australian Wheat. Crop Pasture Sci..

[101] Muterko A., Balashova I., Cockram J., Kalendar R., Sivolap Y. (2015). The New Wheat Vernalization Response Allele *Vrn-D1s* Is Caused by DNA Transposon Insertion in the First Intron. Plant Mol. Biol. Rep..

[102] Shcherban A., Emtseva M., Efremova T. (2012). Molecular Genetical Characterization of Vernalization Genes *Vrn-A1*, *Vrn-B1* and *Vrn-D1* in Spring Wheat Germplasm from Russia and Adjacent Regions. Cereal Res. Commun..

[103] Shcherban A.B., Börner A., Salina E.A. (2015). Effect of *VRN-1* and *PPD-D1* Genes on Heading Time in European Bread Wheat Cultivars. Plant Breed..

[104] Milec Z., Strejčková B., Šafář J. (2023). Contemplation on Wheat Vernalization. Front. Plant Sci..

[105] Mcintosh R.A., Dubcovsky J., Rogers W.J., Morris C.F., Appels R., Xia X.C., Science R., Azul C., Aires P.D.B., Plant M. Catalogue of Gene Symbols for Wheat: 2013–2014 Supplement. Proceedings of the 12th International Wheat Genetics Symposium.

[106] Li G., Yu M., Fang T., Cao S., Carver B.F., Yan L. (2013). Vernalization Requirement Duration in Winter Wheat Is Controlled by *TaVRN-A1* at the Protein Level. Plant J..

[107] Yan L., Li G., Yu M., Fang T., Cao S., Carver B.F. (2015). Genetic Mechanisms of Vernalization Requirement Duration in Winter Wheat Cultivars. Advances in Wheat Genetics: From Genome to Field: Proceedings of the 12th International Wheat Genetics Symposium.

[108] Distelfeld A., Tranquilli G., Li C., Yan L., Dubcovsky J. (2009). Genetic and Molecular Characterization of the VRN2 Loci in Tetraploid Wheat. Plant Physiol..

[109] Nishimura K., Handa H., Mori N., Kawaura K., Kitajima A., Nakazaki T. (2021). Geographical Distribution and Adaptive Variation of VRN-A3 Alleles in Worldwide Polyploid Wheat (*Triticum* spp.) Species Collection. Planta.

[110] Berezhnaya A., Kiseleva A., Leonova I., Salina E. (2021). Allelic Variation Analysis at the Vernalization Response and Photoperiod Genes in Russian Wheat Varieties Identified Two Novel Alleles of *Vrn-B3*. Biomolecules.

[111] Pugsley A.T. (1972). Additional Genes Inhibiting Winter Habit in Wheat. Euphytica.

[112] Katou K. (1993). Chromosomal Location of the Genes for Vernalization Response, *Vrn2* and *Vrn4*, in Common Wheat, *Triticum aestivum* L. Wheat Inf. Serv..

[113] McIntosh R.A., Yamazaki Y., Dubcovsky J., Rogers J.W., Morris C., Appels R., Xia X., Azul B. Catalogue of Gene Symbols for Wheat: 2013–2014. Proceedings of the 12th International Wheat Genetics Symposium.

[114] Xue Q., Xiong H., Zhou C., Guo H., Zhao L., Xie Y., Gu J., Zhao S., Ding Y., Xu L. (2023). Gene Mapping and Identification of a Missense Mutation in One Copy of *VRN-A1* Affects Heading Date Variation in Wheat. Int. J. Mol. Sci..

[115] Royo C., Dreisigacker S., Alfaro C., Ammar K., Villegas D. (2016). Effect of *Ppd-1* Genes on Durum Wheat Flowering Time and Grain Filling Duration in a Wide Range of Latitudes. J. Agric. Sci..

[116] Nazim Ud Dowla M.A.N., Edwards I., O’Hara G., Islam S., Ma W. (2018). Developing Wheat for Improved Yield and Adaptation under a Changing Climate: Optimization of a Few Key Genes. Engineering.

[117] Whittal A., Kaviani M., Graf R., Humphreys G., Navabi A. (2018). Allelic Variation of Vernalization and Photoperiod Response Genes in a Diverse Set of North American High Latitude Winter Wheat Genotypes. PLoS ONE.

[118] Esposito S., D’Agostino N., Taranto F., Sonnante G., Sestili F., Lafiandra D., De Vita P. (2022). Whole-Exome Sequencing of Selected Bread Wheat Recombinant Inbred Lines as a Useful Resource for Allele Mining and Bulked Segregant Analysis. Front. Genet..

[119] Walkowiak S., Gao L., Monat C., Haberer G., Kassa M.T., Brinton J., Ramirez-Gonzalez R.H., Kolodziej M.C., Delorean E., Thambugala D. (2020). Multiple Wheat Genomes Reveal Global Variation in Modern Breeding. Nature.

[120] Annicchiarico P., Nazzicari N., Bouizgaren A., Hayek T., Laouar M., Cornacchione M., Basigalup D., Monterrubio Martin C., Brummer E.C., Pecetti L. (2022). Alfalfa Genomic Selection for Different Stress-Prone Growing Regions. Plant Genome.

[121] Esposito S., Vitale P., Taranto F., Saia S., Pecorella I., D’Agostino N., Rodriguez M., Natoli V., De Vita P. (2023). Simultaneous Improvement of Grain Yield and Grain Protein Concentration in Durum Wheat by Using Association Tests and Weighted GBLUP. Theor. Appl. Genet..

